# Palatability of Selected Alpine Plant Litters for the Decomposer *Lumbricus rubellus* (Lumbricidae)

**DOI:** 10.1371/journal.pone.0045345

**Published:** 2012-09-18

**Authors:** Alexander Rief, Brigitte A. Knapp, Julia Seeber

**Affiliations:** 1 Institute of Ecology, University of Innsbruck, Innsbruck, Austria; 2 Institute of Microbiology, University of Innsbruck, Innsbruck, Austria; University of Leipzig, Germany

## Abstract

On alpine pastureland the decline in large-bodied earthworm numbers and biomass after abandonment of management might be the result of a shift from highly palatable grass litter to poorly digestible leaf litter of dwarf shrubs. To test this hypothesis, we analysed nitrogen, phosphorous and total phenolic contents of fresh and aged litter of eight commonly occuring alpine plant species and compared consumption rates of these food sources in a controlled feeding experiment with *Lumbricus rubellus* (Lumbricidae). Furthermore, we analysed the microbial community structure of aged litter materials to check for a relationship between the microbial characteristics of the different plant litter types and the food choice of earthworms. Plant litters differed significantly in their chemical composition, earthworms, however, showed no preference for any litter species, but generally rejected fresh litter material. Microbial community structures of the litter types were significantly different, but we could find no evidence for selective feeding of *L. rubellus*. We conclude that *L. rubellus* is a widespread, adaptable ubiquist, which is able to feed on a variety of food sources differing in quality and palatability, as long as they have been exposed to wheathering.

## Introduction

Earthworms play an important role in litter decomposition by shredding and comminuting newly accumulated litter material, incorporating it in the mineral soil and thus making it accessible to secondary decomposers and microbes. On managed alpine pastureland earthworms, especially the epigeic/hemiedaphic species *Lumbricus rubellus* HOFFMEISTER, predominate both in abundance and biomass. After abandonment its number decreases rapidly, resulting in reduced litter decomposition rates [1], [2]. Reasons for this decline might be the change in its food supply, shifting from litter of highly palatable annual grasses to poorly digestible leaf litter from perennial grasses and particularly dwarf shrubs within a period of about 10–40 years, depending on the speed of dwarf shrub immigration ([1], Seeber J and Meyer E unpublished data). Studies have shown that earthworm populations are often food limited, the decisive factor is usually the quality rather than the quantity of the food material [3]. The palatability of the litter material for earthworms is influenced by the physical toughness (fibre and lignin content), as well as the chemical defence of the plant species [4]. Various plant traits such as the C:N ratio [5] and the sort and amounts of polyphenols they contain [6] are major contributors to the palatability of leaf litter. An additional decisive factor might be the microbial community growing on the litter material, as it can increase palatability, especially in nutrient-poor litter (as summarized in [3]), as well as pose a valuable food source themselves [7]. Generally, fast growing plants like grasses have high N and P contents with low C:N and C:P ratios and decompose rapidly, whereas plants with slow growth rates like dwarf shrubs are rich in recalcitrant compounds such as lignin and phenolics and exhibit slower litter decomposition rates [4], [8]–[10]. Consequently grasses tend to produce high-quality, nutrient-rich litter while ericaceous plants, such as *Vaccinium gaultheroides* and *Calluna vulgaris* (Ericaceae), produce low-quality, recalcitrant litter [11]. High quality litter stimulates the growth and activity of bacterial-based microbial communities and supports high earthworm densities whereas poor quality litter favours the growth of fungi and high densities of macro- and microarthropods [10], [12].

Although the general importance of plant ingredients for litter palatability to invertebrate decomposers has been widely recognized, only few studies have measured these parameters in litter of different stages, especially for alpine habitats, where due to low decomposition rates variously aged litter is available for soil animals. In the present study we hypothesised that in alpine pastures the decline in earthworm numbers and biomass after abandonment of management might be caused by a shift from highly palatable grass litter to poorly digestible leaf litter of dwarf shrubs. To test this hypothesis we assessed carbon, nitrogen, phosphorous and total polyphenol contents of plant litters of two different decompositional stages and compared consumption rates of these food sources in a controlled feeding experiment with *L. rubellus*. In a second step, we analysed the microbial community structure on the aged litter material to test whether a relationship exists between the plant species’ specific microbial characteristics and the food choice of earthworms, as we hypothesised that *L. rubellus* might actively select food sources harbouring specific microbial communities.

## Materials and Methods

### Ethics Statement

The Institute of Ecology, University of Innsbruck has a general permit to use the Kaserstattalm area for scientific purposes. No endangered or protected species were involved in the experiment.

### Litterbags

Eight plant species belonging to three functional groups (herbs, grasses, dwarf shrubs) and being representative for either managed or abandoned alpine meadows and pastures were chosen. *Dactylis glomerata* (Poaceae) is an abundant grass species on managed meadows and pastures. *Nardus stricta* (Poaceae), *Luzula lutea* (Juncaceae), *Vaccinium myrtillus*, *V. gaultheroides*, *V. vitis-idea* and *Calluna vulgaris* (all Ericaceae) are abundant grasses and dwarf shrubs on abandoned areas. The herb *Trollius europaeus* (Ranunculaceae) can be found in both managed and abandoned areas.

Litter material was collected from a managed and an abandoned alpine cow pasture at the Kaserstattalm (Central Alps, Tyrol, Austria, 11°18′ E, 47°07′ N, 2000 m a.s.l.) in September and October 2006 shortly before leaf fall. Either dead leaves were clipped from living plants (grasses), homogeneously distributed all over the research sites, or fallen leaves were gathered from beneath dwarf shrubs. This procedure was chosen to guarantee species-specific litter material, replicates for analyses were taken from this pooled material.

To evaluate chemical parameters of fresh litter about 20 g of air-dried plant material was dried at 50°C to avoid oxidation by still active enzymes. The remaining material was filled into litterbags (30×20 cm, mesh size: 102 µm, about 40–50 g air-dried litter per bag, one bag per species for chemical and microbiological analyses, one bag for feeding experiments). On an abandoned alpine pasture on the Kaserstattalm the vegetation was cut and the litterbags were fixed to the ground with the aid of tent pegs to guarantee close contact with the soil. In October 2007 the litterbags were sampled to analyse one year-old (henceforth referred to as aged) litter material. Sample material was again dried at 50°C.

### Analysis of Total Polyphenolics

Dried litter material was ground with a ball mill (3 replicates of each litter type per sampling date). Phenolic compounds were extracted with 50% ethanol, the extract was filtered through a glass microfibre filter [13]. The filtrate was then used for phenolic analyses.

For the quantification of total phenolics from plant materials, the Folin-Ciocalteau method, a general phenolic assay, was applied, following the prodecure outlined in Torres et al. [14]. Commercially available gallic acid was used as standard. The absorption was photometrically measured.

### Analysis of Total Phosphorous

Dried and milled litter material (3 replicates of each litter type per sampling date) was analysed by a high ignition method proposed by Illmer [15]. Material was incinerated at 550°C, extracted with 0.5 M H_2_SO_4_ and filtered through phosphate-free filters. For determination the molybdenum blue procedure described by Olsen and Sommers [16] was used.

### Analysis of C- and N-contents

Dried and milled litter material was weighed into tin capsules (3 replicates of each litter type per sampling date) and analysed by a C-H-N analyser (Flash EA 1112 NC Analyzer, ThermoFisher Scientific).

### Feeding Experiments

**Figure 1:**
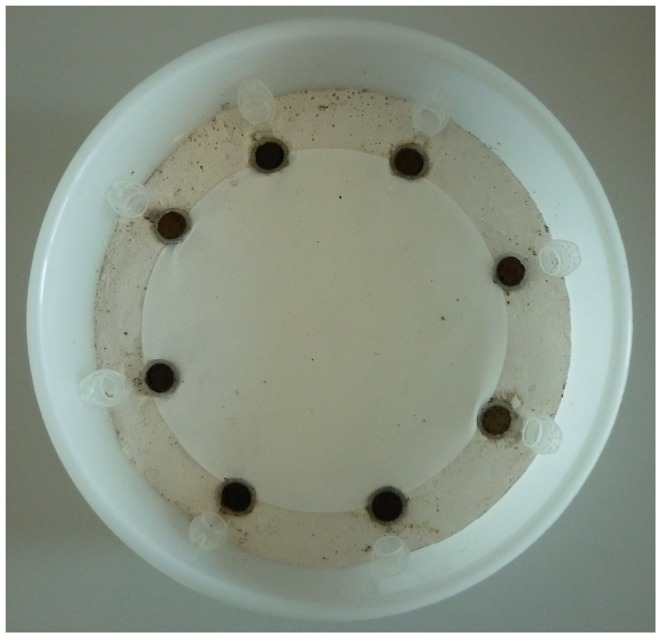
Experimental setup of the feeding chambers. Plastic boxes (diameter 18 cm, height 8 cm) filled with gypsum (4 cm) and eight microcentrifuge tubes containing litter material (one for each plant species, randomly placed).

Specimens of *L. rubellus* were handcollected at the research site to specifically investigate alpine exemplars that are naturally subjected to the conditions following land-use changes. Animals were kept in plastic boxes containing a mixture of soil and cow dung, an optimal and naturally occurring food source for earthworms, taken from the same site, in a climatic chamber (15°C) for about one month until start of the experiment. Foodchoice chambers were adapted to the characteristic behavior patterns and preferences of Lumbricidae. Circular plastic boxes (diameter 18 cm, height 8 cm) were filled with gypsum (4 cm) and eight microcentrifuge tubes (1.5 ml, one for each plant species) were inserted along the outer margin so that earthworms had easy access to the food without being able to remove the material from the tubes (Fig. 1). The slightly rough surface of the gypsum was covered with a permeable film. The gypsum was moistenend and two layers of damp filter paper (Whatman Nr. 1, 12.5 cm) were placed upon it to guarantee humid conditions for earthworms. The covers of the boxes contained small holes for aeration. Earthworms were left starving 24 h prior to the experiment.

Fresh and aged litters of all eight plant species were used as food sources for the experiment. As we focused on identifying chemical and microbiological effects, plant material was milled to exclude possible physical parameters (e.g. roughness of leaves). Dried material was weighed and mixed with water to a thick paste. Prior to insertion of the food source a cling-film was placed into the tubes to form a plug, thus ensuring that earthworms could not use the tube as a shelter. Each tube was then filled with moist food material (dry weight about 70 mg) and placed into the holes in the bottom of the tray. The tubes were placed at equal distances from each other with random positioning to the neighbouring food types.

Two sets of feeding chambers were prepared, each with 10 replicates: one set with fresh litter and one set with aged litter material. Earthworms were inserted individually in the middle of the boxes to allow them to move freely und unbiasedly. Feeding chambers were closed tightly and held in a climatic chamber (15°C) for the entire experiment. To avoid mouldiness, tubes were removed after three days and food was dried and weighed, new tubes were inserted. After six days the cumulative decrease in weight was measured. The filter paper was replaced regularly to prevent earthworms from feeding on their faeces.

### Microbial Community

The microbial community structure was analysed for aged litter material only, as the feeding experiments had already shown that earthworms reject fresh litter material as food source.

Four samples of each litter type were analysed. The replicates come from a pool taken from a large sampling area, therefore exemplifying a representative sample for each litter type. Furthermore, all replicates have been treated separately from DNA extraction to DGGE analysis. DNA extraction, amplification of target DNA and DGGE analyses have been described in detail in Knapp et al. [17]. In short, DNA extraction from the litter samples was performed using the PowerSoil DNA Isolation Kit (MoBio Laboratories Inc., USA). Extracted DNA was amplified in a PCR thermocycler (PCR Express, ThermoHybaid, Germany) using the universal 16S rRNA primer set 63f (CAG GCC TAA CAC ATG CAA GTC) [18] and 1378r (CGG TGT GTA CAA GGC CCG GGA ACG) [19] to amplify total bacterial communities. For the amplification of the fungal 18S rRNA, primers FR1GC (CCC CCG CCG CGC GCG GCG GGC GGG GCG GGG GCA CGG GCC GAI CCA TTC AAT CGG TAI T) and FF390 (CGA TAA CGA ACG AGA CCT) [20] were chosen. For PCR conditions see Knapp et al. [17].

After amplification PCR products were checked by electrophoresis in 1.5% (w/v) agarose gels and ethidium bromide staining (10 mg⋅mL^−1^), as well as quantified using PicoGreen dsDNA quantitation reagent (Invitrogen, USA).

Denaturing gradient gel electrophoresis (DGGE) was performed with the Ingeny PhorU2 system (Ingeny International BV, The Netherlands). For bacterial DGGE, 2 µL PCR product was loaded on to 8% (w/v) polyacrylamide gels with a denaturing gradient of 40% to 70% (100% denaturant according to 7 M urea plus 40% formamide in 1X TAE-buffer) and was run for 16 h at 100 V and at a constant temperature of 60°C in 1x TAE-buffer (pH 7.4). For fungal amplicons, 7% (w/v) polyacrylamide gels with a denaturing gradient of 30–60% were prepared. After electrophoresis, the gels were stained with silver nitrate using an automated gel stainer (Amersham Pharmacia Biotech, Germany), photographed and air dried for storage.

### Statistical Analyses

Differences in chemical parameters between plant species, woody and herbaceous plants as well as between fresh and aged litter were analysed separately by one-way ANOVA. Multivariate analyses of differences in polyphenol, phosphorous and nitrogen contents (dependent variables) between litter types of different ages (independent variables plant species and litter age) were analysed by MANOVA. Consumption rates were compared by two-way ANOVA (factors plant type and litter age). Statistical analyses were performed using STATISTICA (StatSoft, Inc. 2007). Effects of chemical parameters on consumption rates were analysed by CANOCO for Windows 4.5 [21]. Canonical correspondence analysis (CCA) was used to examine how the consumption rates of *L. rubellus* were related to chemical parameters of plants. Statistical significance of axes was tested using Monte Carlo permutation tests (999 permutations under the reduced model).

DGGE banding patterns were normalized and analysed using the GelCompar II software package, version 4.0 (Applied Maths, Ghent, Belgium). Cluster analyses (ward-method, squared euclidean distances) were performed to evaluate similarities between the microbial communities attached to different litter species using STATISTICA.

## Results

### Chemical Parameters

Fresh litter material showed distinctly higher contents of total phenolics in dwarf shrubs than in herbaceous plants (F_1_,_22_ = 57.10, p<0.001), differences between plant species were highly significant (F_7,16_ = 61.55, p<0.001) (Table 1). Total phenolics strongly decreased during decomposition (F_1,46_ = 42.21, p<0.001), however, differences between plant species were less pronounced (F_7,16_ = 3.58, p = 0.017). Differences between woody and herbaceous plants remained significant (F_1,22_ = 30.17, p<0.001).

**Table 1:** Chemical parameters of fresh and aged litter material.

Species	Litter type	P_total_	C_total_	N_total_	Phenolics_total_
		mg g^−1^	mg g^−1^	mg g^−1^	mg/l GAE
*Vaccinium gaultheroides*	fresh	1.95 (0.012)	496 (12)	10.0 (0.82)	574 (76)
*V. myrtillus*	fresh	2.06 (0.031)	489 (61)	10.3 (0.86)	556 (10)
*V. vitis-idea*	fresh	0.82 (0.134)	543 (12)	15.4 (0.97)	654 (31)
*Calluna vulgaris*	fresh	1.11 (0.147)	513 (8)	15.7 (0.58)	278 (47)
*Trollius europeus*	fresh	1.78 (0.130)	421 (25)	17.5 (0.99)	227 (14)
*Dactylis glomerata*	fresh	1.83 (0.255)	417 (30)	26.2 (1.35)	79 (24)
*Nardus stricta*	fresh	1.52 (0.220)	447 (4)	17.1 (0.31)	120 (5)
*Luzula lutea*	fresh	2.08 (0.103)	420 (19)	11.7 (0.19)	153 (6)
*Vaccinium gaultheroides*	aged	2.84 (0.035)	488 (23)	32.6 (0.99)	49 (47)
*V. myrtillus*	aged	2.65 (0.036)	506 (11)	26.7 (1.09)	55 (14)
*V. vitis-idea*	aged	2.01 (0.073)	534 (13)	17.0 (0.56)	50 (3)
*Calluna vulgaris*	aged	2.52 (0.039)	503 (30)	22.8 (1.74)	56 (21)
*Trollius europeus*	aged	2.88 (0.019)	443 (6)	32.5 (0.82)	18 (13)
*Dactylis glomerata*	aged	3.01 (0.065)	447 (7)	23.6 (1.38)	5.7 (3)
*Nardus stricta*	aged	2.80 (0.037)	458 (15)	29.5 (0.95)	8.1 (7)
*Luzula lutea*	aged	2.83 (0.060)	476 (6)	14.5 (0.84)	18 (2)

Mean value with standard deviation in parentheses, n = 3.

Plant species differed highly significantly in their phosphorous and nitrogen contents, both in fresh (P F_7,16_ = 28.02, p<0.001; N F_7,16_ = 116.34, p<0.001) and in older litter material (P F_7,15_ = 101.09, p<0.001; N F_7,16_ = 110.88, p<0.001). Total phosphorus and nitrogen increased with decomposition time (P F_1,45_ = 95.48, p<0.001; N F_1,46_ = 31.41, p<0.001), while for carbon almost unnoticeable shifts were detectable (F_1,46_ = 1,13, p = 0.29). Consequently C:N ratio decreased with decomposition time. Nitrogen contents in fresh litter differed significantly between woody and herbaceous plants (F_1,22_ = 8.76, p = 0.007), while they equalled in aged litter (F_1,22_ = 0,01, p = 0.94). For phosphorous content it was contrary, significant differences could only be detected in aged litter (F_1,21_ = 13.43, p = 0.002).

Multivariate analyses of polyphenolic, phosphorous and nitrogen contents showed highly significant differences between litter types in both decompositional stages (Wilks Lamda = 0.002, p<0.001).

### Feeding Preferences

**Figure 2:**
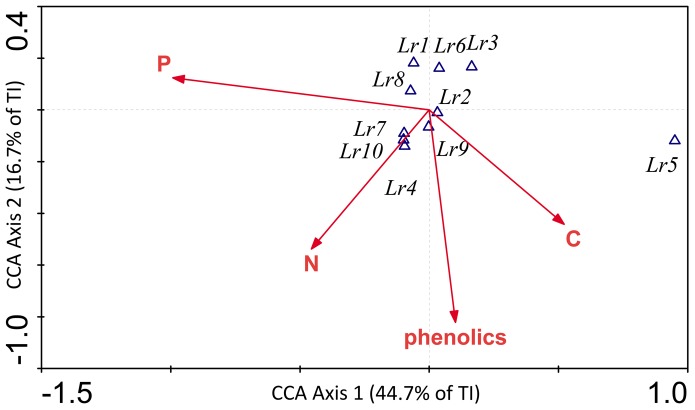
Canonical correspondence analysis (CCA) of chemical parameters of litter and individual consumption rates of *L. rubellus*. Arrows…litter, triangles…consumption rates of *L. rubellus*. Eigenvalues axis 1∶0.546 (F = 2.43, p = 0.026), axis 2∶0.203. Variation explained by axes is given as a percentage of total variation (total inertia, TI).

There was an obvious preference of *L. rubellus* for aged litter, feeding on fresh litter material was very limited (F_1,7_ = 21.49, p<0.001). This was also noticeable as four earthworms from the feeding experiment with fresh litter material did not survive. However, no feeding preferences for litter of any plant species could be detected (F_1,7_ = 1.32, p = 0.247), *L. rubellus* clearly fed on all available food sources in very different amounts (as indicated by rather high standard deviations), as long as they had undergone a period of weathering (Table 2). Analyses of feeding preferences based on functional groups (herbaceous and woody plants) did also not show any significances (F_1,126_ = 0.99, p = 0.323). Furthermore, no effect of chemical parameters on consumption rates could be verified (Fig. 2).

**Table 2:** Consumption rates of different litter types of *L. rubellus.*

Species	Consumption (mg/ind)	
	fresh litter	aged litter
*Vaccinium gaultheroides*	5.38 (5.12)	32.91 (28.78)
*V. myrtillus*	4.24 (8.62)	12.80 (21.59)
*V. vitis-idea*	0.71 (2.04)	10.62 (14.48)
*Calluna vulgaris*	0.83 (2.36)	17.41 (19.20)
*Trollius europeus*	0.80 (2.28)	12.52 (12.44)
*Dactylis glomerata*	8.12 (10.23)	9.88 (10.82)
*Nardus stricta*	0.57 (1.63)	12.77 (12.73)
*Luzula lutea*	3.13 (6.65)	19.23 (16.76)

Mean values with standard deviation in parentheses (fresh litter: n = 6, aged litter: n = 10).

### Microbial Community

**Figure 3:**
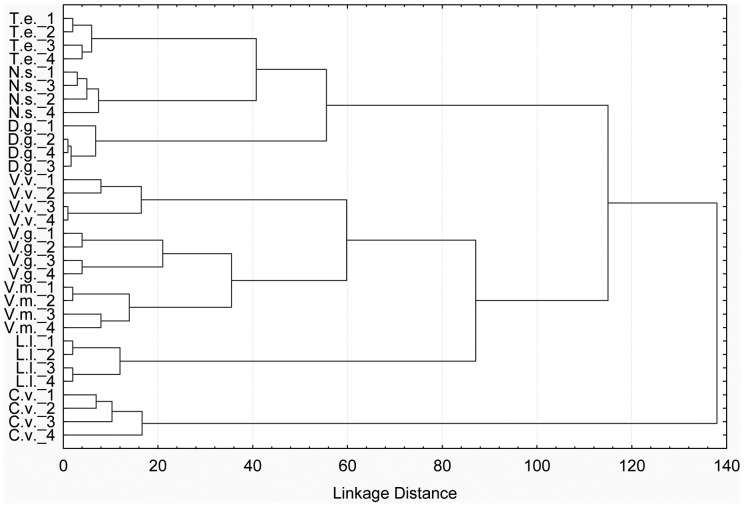
Cluster analyses of similarities between bacterial and fungal communities attached to litter species. V.g. *Vaccinium gaultheroides*, V.m. *V. myrtillus*, V.v. *V. vitis-idea*, C.v. *Calluna vulgaris*, T.e. *Trollius europaeus*, D.g. *Dactylis glomerata*, N.s. *Nardus stricta*, L.l. *Luzula lutea*, n = 4.

Bacterial and fungal fingerprints showed a complex microbial community attached to the plant litter, with some bands common to all litter types as well as with bands characteristic for each of the different kinds of litter. Cluster analyses of the microbial fingerprints separated the eight plant species very clearly (Fig. 3) and it can be inferred from the graph that each litter species harbours a distinct microbial community. Independend of whether bacterial and fungal communities were analysed separately or in combination, the analysis resulted in almost identical dendrogramms with three main clusters: (1) the Poaceae *D. glomerata* and *N. stricta* plus the herb *T. europaeus*, (2) the dwarf shrubs *Vaccinium spp.* together with Juncaceae *L. lutea* and (3) *C. vulgaris* forming a sole cluster.

## Discussion

We presented results on changes in litter chemical parameters with decomposition time of alpine plant species belonging to different functional groups. Their association with consumption rates of the earthworm *Lumbricus rubellus*, an important decomposer species on managed and abandoned alpine pastureland was analysed. The chemical traits of litter (N, P and total phenolics concentrations) are frequently used to evaluate the quality and palatability of litter for decomposer species (e.g. [5], [22], [23]). Our results showed that, although the plant species differ significantly in their chemical composition and thus in their nutritional quality, no feeding preferences for any litter type could be observed, except a clear preference for aged litter material.

Phenolic compounds are usually of plant origin and play an important ecological role by directly or indirectly influencing numerous living organisms [24], [25] and soil processes through inhibiting nitrification, decomposition and nutrient recycling [26]. It has been found that especially tannins act as feeding deterrents, as earthworms and other soil animals do not possess digestive enzymes to process phenolics, their degradation is essentially due to the activities of enzymes produced by the gut bacteria [27]. In our study total phenolics decreased strongly within one year, most of this loss of phenolic compounds is probably caused by leaching [28]. In fresh litter material the content of total phenolics is distinctly higher in dwarf shrubs than in grass plants, woody plants usually contain higher molecular weight polyphenolic compounds than herbaceous [10], [29]. However, these differences wear off rapidly as decomposition continues. Only *Vaccinium vitis-idea* having relatively thick and rough leaves shows a slightly delayed phenolic degradation. Consequently, the importance of phenolic compounds for litter palatability declines with gradual decomposition. Additionally, polyphenols can form complexes with proteins, which are not digestible for most decomposer species save earthworms, which are able to directly use N originating from these protein-polyphenol complexes [26].

Besides polyphenols, nitrogen and phosphorous contents are useful indicators for palatability when comparing the food quality of different types of litter [3], [23]. Low C:N and C:P ratios support N and P mineralization by the microbial community [10] and might therefore provide macro-decomposers with more nutrients. In our study both nitrogen and phosphorous contents increased relatively with decomposition time, resulting in lower C:N and C:P ratios. David and Handa [30] already explained the increase in palatability with the decrease in C:N in more decomposed litter. The reduced polyphenol contents and the low C:N and C:P ratios in aged litter material seem to have been the crucial reasons why it was primarily consumed by *L. rubellus* compared with fresh litter in our study. The preference for aged litter material has generally been reported for detritivorous soil animals (e.g. [3], [31]: earthworms; [30], [32]: millipedes and woodlice).

For carbon almost unnoticeable shifts were detectable. Studies on the mass-to-carbon ratio, meaning that C would be lost from the litterbag at the same relative rate as the mass, has shown that this assumption does not always hold. Zhang et al. [33] have explained this with the fact that C and non-C fractions are decaying at about the same relative rates, which means that C concentrations remain constant during decomposition. Similar results have, e.g., been shown by Berg et al. [34].

In our experiment we analysed the microbial community structure on the aged litter material, DGGE analyses showed significant differences between the food sources. Although it is generally known that earthworms ingest microbes growing on the litter material, to our knowledge it has never been investigated whether the composition of these microbial communities is a factor for food selection. Although fungi, bacteria and also protozoans form an important part of the diet of earthworms [7], [35], only limited evidence exists that earthworms actively discriminate between certain microbes [36]. In a laboratory food choice experiment Bonkowski et al. [37] have shown that *L. rubellus* has strong feeding preferences for certain fungal species, for bacteria this has only been proven for isopods [36], [38], [39]. As it has been shown that the microbial community in the gut of earthworms is diet-related [40], [41] and therefore may support the adaptation of these animals to new dietary conditions after abandonment of alpine pastures, we hypothesised that *L. rubellus* actively selects food sources harbouring specific microbial communities. Although we found no evidence to support this hypothesis from our laboratory experiment, detailed structural and functional analyses of the bacterial and fungal communities on the litter materials are urgently needed to further investigate this question. Especially field data and data on litter mixtures will help to evaluate the relationship between macro-decomposers and litter-colonising microbiota in terms of feeding preferences.

Food choice experiments have been widely used to investigate feeding preferences of earthworms. Several studies reported selective feeding of epigeic earthworms while endogeic species used a broader dietary spectrum [5], [31], [37], [42]. From the results of our feeding experiment we conclude that *L. rubellus,* although a detritivorous species, is a generalist feeder as it fed on all aged litter types in varying amounts (expressed by extremely high standard deviations). Our main hypothesis that the poor quality of dwarf shrub litter is the main reason for large-bodied earthworms such as *L. rubellus* to retreat after abandonment did not prove true. Phenolic compounds, which are reported to have higher concentrations in woody plants than in grasses and herbs [10], [29], have been named as strong feeding deterrents for soil animals [27]. We showed that dwarf shrubs indeed contain higher amounts of phenolics than deciduous plants, but that the differences are only marginal in aged compared to fresh litter material. *L. rubellus* did not refrain from feeding on any aged plant material due to its amount of phenolic compounds (Fig. 2). Furthermore, the C:N and C:P ratios generally decreased with decomposition time, thus making almost all litter materials better palatable, with the amelioration being more pronounced in dwarf shrubs (e.g. *V. gaultheroides* C:N of 50 in fresh to 15 in aged litter) than in grasses (e.g. *N. stricta* C:N of 26 in fresh to 15.5 in aged litter). Our second hypothesis that the earthworm selects its food source according to a specific microbial community structure attached to the litter did also not hold true despite the fact that each litter species harbours a specific microbial community.

We therefore conclude that as adaptable and widespread ubiquist which occupies a variety of habitats *L. rubellus* seems to be able to digest food sources differing in quality and palatability, as long as it has been exposed to weathering. This is supported by results of laboratory feeding experiments and in a field study using labelled ^15^N as a tracer in which it was shown that dwarf shrub litter represents a highly potential food source for earthworms on alpine pastureland [2], [43]. Therefore, other factors than the quality and palatability of the food sources must be the decisive reasons for the decline of *L. rubellus* after abandonment.
